# Smoking is predictive of poorer distant metastasis-free and progression free-survival in soft tissue sarcoma patients treated with pre-operative radiotherapy or chemoradiotherapy

**DOI:** 10.1186/s13569-018-0088-8

**Published:** 2018-04-16

**Authors:** Nicholas P. Gannon, David M. King, Manpreet Bedi

**Affiliations:** 1 Department of Orthopaedic Surgery, Medical College of Wisconsin, Milwaukee, WI 53226 USA; 2 Department of Radiation Oncology, Medical College of Wisconsin, 9200 West Wisconsin Ave, Milwaukee, WI 53226 USA

**Keywords:** Cancer, Smoking, Nicotine, Metastatic survival, Prognosis

## Abstract

**Background:**

Soft tissue sarcomas (STS) are often treated with pre-operative radiation (RT), with or without chemotherapy, followed by wide local excision. Prognosis for these patients involves an interplay of tumor and patient characteristics. Known prognostic determinants include tumor size, grade, response to therapy, and patient characteristics such as age. While smoking is negatively correlated with outcomes in various malignancies, the impact on STS is unknown. We aimed to assess if smoking impacts overall (OS), distant metastasis-free (DMFS), and progression-free (PFS) survival in patients with STS treated with pre-operative RT.

**Methods:**

Between 2000 and 2015, 166 patients with STS were identified from our prospective database. Patient variables were retrospectively reviewed. Smoking was defined as a ≥ 10 pack year history of current and former smokers. Survival was evaluated using the fisher exact test for univariate (UVA) and logistic regression for multivariate (MVA) analysis.

**Results:**

Fifty-seven (34.3%) patients had smoking histories of ≥ 10 pack years. On UVA, smoking was associated with decreased DMFS (p = 0.0009) and PFS (p = 0.0036), but not OS (p = 0.05). Smoking held significance on MVA for both DMFS and PFS. Current smokers and patients with ≥ 24-month follow-up demonstrated decreased DMFS and PFS on UVA and MVA.

**Conclusions:**

Current smokers and patients with a significant smoking history demonstrated decreased DMFS and PFS in STS patients treated with pre-operative RT. Smoking may cause immunologic compromise and therefore lead to higher rates of progression and distant metastasis.

## Background

Soft tissue sarcomas (STS) are rare, heterogeneous solid tumors of mesenchymal origin. Approximately 12,000 new cases of STS are diagnosed per year in the United States with a mortality rate of 40% [1]. Historical standard-of-care treatment has included extremity amputation in an effort to reduce recurrence rates and improve survival. However, there has been an evolution towards limb-salvage therapy with wide local excision and radiation (RT) with or without chemotherapy. Pre-operative RT followed by wide local excision has been widely accepted as appropriate management and leads to excellent rates of local control and survival [2–10].

Despite excellent local control rates, many patients with STS develop metastases and succumb to their disease. Factors associated with survival in STS include inherent tumor biology, response to therapy, and patient characteristics. Similar findings have been shown when assessing overall survival (OS), local recurrence, distant recurrence, or post-metastasis survival [11–14]. While the previous investigations included large cohorts and reported consistently reproducible results, many did not analyze patient lifestyle behaviors with survival outcomes.

Smoking has been strongly correlated with the pathogenesis of many malignancies including, but not limited to, head and neck, lung, and bladder cancer [15], and smoking has been shown to impact disease outcomes in these cancers. Studies have also demonstrated increased cancer mortality risks amongst both men and women, current and former smokers [16, 17]. Shopland et al. projected that smoking leads to 21.5% of cancer related deaths in women and 45% of cancer related deaths in men [17].

Contrary to other malignancies, there has been no direct link demonstrated between smoking and the incidence of STS. Similarly, sparse information exists on the significance of smoking associated survival in STS. The investigations that have gathered/reported smoking history and correlated it with various measures of survival conclude no significance [18–20].

In the present study, we retrospectively analyzed a cohort of STS patients treated with pre-operative RT or chemoradiotherapy (CRT) followed by limb-sparing surgery. The primary objective of this investigation was to assess if smoking correlates with OS, distant metastasis-free survival (DMFS), and progression-free survival (PFS).

## Methods

This research was reviewed and approved by the Medical College of Wisconsin Institutional Review Board (IRB) and all investigators completed training in both human research and patient privacy.

### Patient population

All patients with primary STS of the extremity and trunk who received pre-operative RT with or without pre-operative chemotherapy followed by surgical resection between November 2000 and August 2015 were reviewed from a prospectively collected database. Patients were staged according to the 2010 American Joint Committee on Cancer (AJCC) system seventh edition. Exclusion criteria included metastatic disease on initial presentation, age < 18 years old, STS of locations other than the extremity or trunk, post-operative RT, recurrent sarcomas, and histopathologic types demonstrating rhabdomyosarcoma, extraosseous primitive neuroectodermal tumor, Ewing’s sarcoma, osteosarcoma, Kaposi’s sarcoma, angiosarcoma, aggressive fibromatosis, or dermatofibrosarcoma protuberans. Patients who did not have complete medical records including treatment information and a pathology report, and follow-up of less than 6 months were also excluded. Patient demographic, clinical, and pathological information were recorded.

### Treatment

All patients with STS were discussed at a multi-disciplinary sarcoma board consisting of surgical and musculoskeletal oncologists, medical and radiation oncologists, musculoskeletal radiologists, and pathologists with specialty training in bone and soft tissue pathology. Tumor board treatment recommendations were presented and discussed with the patient.

### Radiation and chemotherapy

All patients were administered pre-operative RT. Patients received a median dose of 50 gray (Gy) using 3D-conformal radiation or intensity-modulated RT (IMRT).

Chemotherapy was discussed and offered to patients who were aged < 70 years, with large (> 5 cm), deep, and high-grade lesions. Chemotherapy was delivered prior to initiation of radiation therapy, using combination doxorubicin–ifosfamide for 1–3 cycles, dependent on patient tolerance and clinical efficacy.

### Surgery

Limb-salvage resection was performed 4–6 weeks following RT. Surgery was grossly approached through normal tissue planes and included sacrifice of tumor-violated arteries and veins. Neurovascular structures were preserved whenever possible. Surgical goals were to achieve negative margins (R0). Vascular and reconstructive plastic surgeons were consulted and involved in cases with difficult wound closures, specifically those requiring free or rotational tissue transfer.

### Statistical analyses

Following exclusion criteria, the sample size for this analysis included 166 patients with available smoking history, defined as a binary variable. Patients who had at least a 10 pack year (20 cigarettes/day/year × 10 years) history of smoking were deemed as smokers, a routinely employed threshold by the oncologic community and former literature. OS, DMFS, PFS rates were estimated using the Kaplan–Meier estimate of the survival function. The log-rank test was used to compare two survival curves. The Fisher exact test was used for univariate (UVA) and a logistic regression analysis was used for multivariate analysis (MVA). For all analyses, type I error was maintained at 0.05 and all tests were two-sided. All statistical analyses were performed using MedCalc (Version 15.6, MedCalc Software bvba, Ostend, Belgium).

## Results

### Patient characteristics

A total of 166 patients received pre-operative RT with or without chemotherapy followed by surgical resection at our institution and were eligible for study inclusion. Of the 57 patients with smoking history, 23 patients were current smokers. Management of patients with positive smoking history is included in Table 1. The median age at diagnosis for the smoking and non-smoking groups were 60 and 55, respectively. The median follow-up was 3.8 years. Subset analysis was performed on 119 patients having a follow-up of ≥ 24 months, 14 patients being smokers. Patient, tumor, and treatment information is summarized in Table 2.

**Table 1:** Management of positive smoking history

	OS	DMFS	PFS	Total
Chemotherapy alone	0	0	0	0
Radiotherapy alone	8	16	15	39
Chemoradiotherapy	11	9	9	29

**Table 2:** Patient, tumor, and treatment characteristics

	Number of patients (%)
	166
Median age	Smoking: 60 Non-smoking: 55
Smoking	No: 109 (65.6) Yes: 57 (34.4)
Current smoker	No: 143 (86.1) Yes: 23 (13.9)
Cardiovascular disease	No: 143 (86.1) Yes: 23 (13.9)
Lung disease	No: 147 (88.6) COPD: 18 (10.8) Primary malignancy: 1 (0.6)
Diabetes mellitus	No: 145 (87.3) Yes: 21 (12.7)
Grade	Low: 16 (9.6) Intermediate: 50 (30.1) High: 100 (60.2)
Histology	Undifferentiated/MFH: 45 (27.1) Liposarcoma: 33 (19.9) Synovial cell: 33 (19.9) Myxofibrosarcoma: 23 (13.9) Leiomyosarcoma: 15 (9.0) MPNST: 6 (3.6) Extraskeletal myxoid chondrosarcoma: 4 (2.4) Fibrosarcoma: 3 (1.8) Solitary fibrous: 3 (1.8) Clear cell: 1 (0.6)
Stage	I: 24 (14.4) II: 25 (15.1) III: 117 (70.5)
Karnofsky performance status	80–100: 141 (84.9) ≤ 70: 25 (15.1)
Tumor location	Upper extremity: 39 (23.5) Lower extremity: 127 (76.5)
Distant lung metastasis	42 (25.3)
Tumor size	< 10 cm: 94 (56.6) ≥ 10 cm: 72 (43.4)
Chemotherapy	No: 107 (64.5) Yes: 59 (35.5)

### Outcomes

**Fig. 1:**
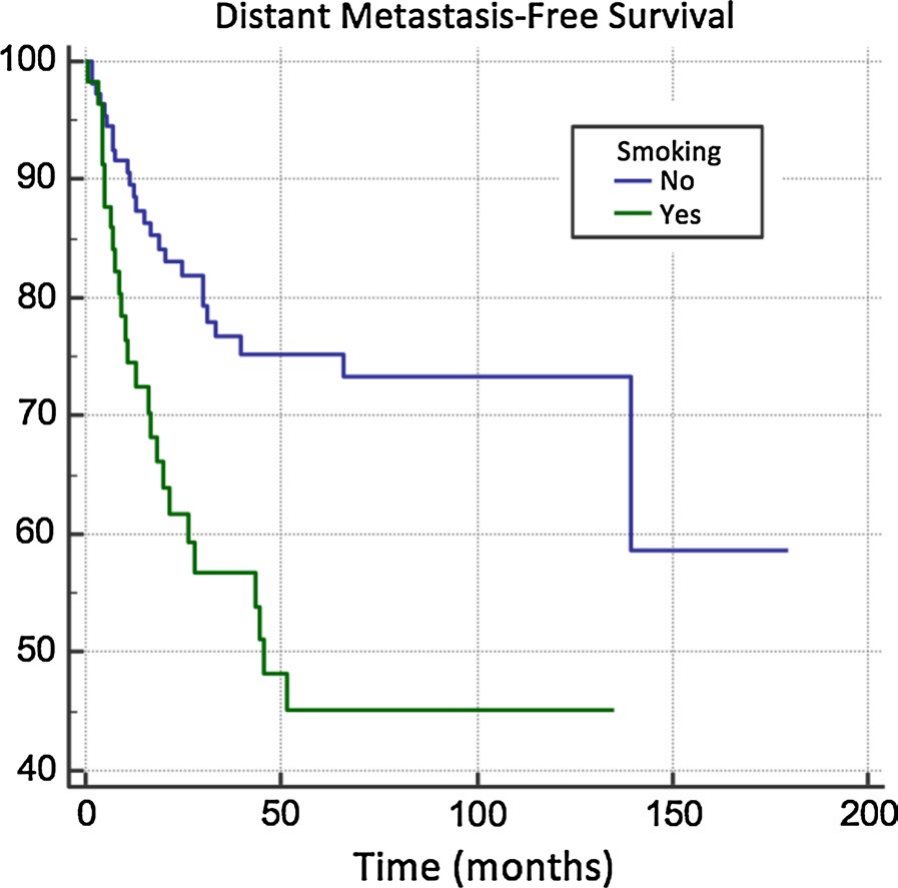
Distant metastasis-free survival and smoking

**Fig. 2:**
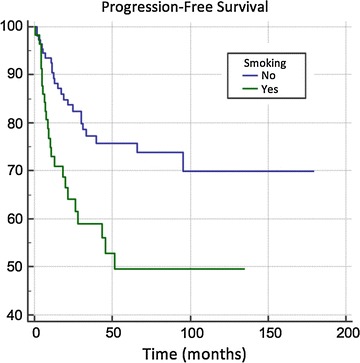
Progression-free survival and smoking

We first looked at all patients following exclusion criteria (≥ 6 month follow up). Smoking trended towards significance for OS on UVA (p = 0.05) and MVA (p = 0.06). Patients with at least a 10 pack year history of smoking had worse DMFS on UVA (p = 0.0009) and MVA (p = 0.0038, 95% CI 1.51–5.14) as well as worse PFS on UVA (p = 0.0036) and MVA (p = 0.0151, 95% CI 1.37–4.49). The median DMFS for non-smokers was not met versus 44.75 months for patients who were smokers (Fig. 1). The median PFS for non-smokers was not met versus 49 months for smokers (Fig. 2).

Analysis of a subset group of patients with ≥ 24-month follow-up revealed that smoking impacted DMFS (p < 0.0001) and PFS (p = 0.0004) on UVA, and DMFS (p = 0.0001, 95% CI 2.14–9.61) and PFS (p = 0.005, 95% CI 1.45–8.21) on MVA.

We further analyzed a second subset of patients who were current smokers at the time of treatment. Current smoking impacted DMFS on UVA (p = 0.0005) and MVA (p = 0.0009, 95% CI 1.62–6.50), and PFS on UVA (p = 0.0014) and MVA (p = 0.0109, 95% CI 1.24–5.09).

In addition to smoking, variables significant for the entire group for DMFS on MVA were age at diagnosis (p = 0.0218, 95% CI 1.01–1.04), tumor size ≥ 10 cm (p = 0.0088, 95% CI 1.56–5.18), low to intermediate grade disease (p = 0.04, 95% CI 0.16–0.99), and a synovial sarcoma histology (p = 0.02, 95% CI 1.04–12.6). Tumor size ≥ 10 cm (p = 0.0012, 95% CI 1.49–5.12) and tumor location of lower extremity (p = 0.0116, 95% CI 1.11–4.87) were the only other significant variables for PFS on MVA in addition to smoking. Variables significant on UVA for OS, PFS and DMFS are located in Table 3.

**Table 3:** UVA for overall, distant-metastasis free, and progression-free survival

Variable	p-value		
	OS	DMFS	PFS
Age	0.0005	0.0218	NS
Smoking	0.05	0.0038	0.0151
Cardiovascular disease	NS	NS	NS
Diabetes	NS	NS	NS
High-grade	0.04	0.04	NS
Histology	NS	0.02	NS
Karnofsky performance status	NS	NS	NS
Tumor location	0.03	NS	0.0116
Tumor size	0.008	0.0088	0.0012
Chemotherapy	NS	NS	NS

*NS* not-significant

## Discussion

Many variables are associated with outcomes in STS treated with pre-operative RT with or without chemotherapy followed by limb-salvage surgery. Factors demonstrated in previous reports include response to treatment, primary tumor biology (grade), tumor size, and individual patient characteristics, such as age. Previous studies have demonstrated that smoking does not seem to be a significant risk factor in the development of STS. Our data, however, indicates that a significant smoking history may increase the risk of patients with STS developing metastatic disease.

Consistent with previous findings, the current study found smoking does not appear to influence OS despite a trend towards significance, perhaps a product of our modest cohort size, lessened statistical power, or short follow-up time. Contrary to other studies, we show cigarette use of at least 10 years was associated with decreased DMFS and PFS on UVA and MVA. Alamanda et al. conducted a 5-year retrospective cohort study using 397 patients with extremity STS and found smoking non-predictive of sarcoma-specific death (p = 0.59), distant metastasis (p = 0.9323), or local recurrence (p = 0.5451) [18]. The authors also noted obese patients had a higher prevalence of smoking than non-obese patients, having no adverse effect on survival [18]. The risk of developing metastatic disease at 6 months in STS is much lower than at 2–3 years [21]. Here we show DMFS and PFS is similar for those patients with ≥ 6- and ≥ 24-month follow-up, as well as current smokers. Behnke et al. demonstrated that smoking status does not contribute to malignant course of disease nor is it correlated with postoperative infection after STS resection [19]. Similarly, smoking history of seventy-seven patients with STS of the thigh provided little prognostic value for local recurrence or distant metastasis, but was associated with an increased risk of death in patients aged less than 50 years, leading authors to conclude smoking prematurely ages STS patients prognostically [20]. This investigation also found increasing age is an independent prognostic indicator for STS patients.

Our cohort selectively included those patients having received only pre-operative RT or CRT, possibly producing conflicting results with those investigations that have concluded smoking has no correlation with all measures of survival [18–20]. Survival differences may contrast from those previous studies which included all patients regardless of treatment course (pre-operative, post-operative RT). The use of neoadjuvant chemotherapy has been shown to increase DMFS in patients < 70 years old with clinical stage III disease of the trunk and extremity [22], although the multimodality synergy of combination RT and chemotherapy remains unknown. At our institution, neoadjuvant chemotherapy is recommended in deep STS that are high grade and ≥ 5 cm in size. In addition, chemotherapy is recommended to select histologies including synovial sarcoma, leiomyosarcoma, myxoid and dedifferentiated liposarcoma, and poorly differentiated sarcoma. No single factor should determine the decision to administer neoadjuvant chemotherapy, and all patients should be discussed at a multidisciplinary sarcoma tumor board. The authors recommend several factors be considered prior to chemotherapy administration including depth, grade, size, and histology. Other factors such as patient preference, significant medical comorbidities, performance status, and positive smoking history should also be evaluated. Additional explanations for discrepancies between our study and those aforementioned are the specific differences in smoking habits/histories, genetic predisposition, and socioeconomic status between the populations examined, or how smoking status was experimentally defined (≥ 10 pack years in the current study).

Although the connection between smoking and disease progression is suggested in our data, the exact mechanism by which smoking increases the risk of disease progression is poorly understood. The present investigation suggests the potential influence of smoking on the immunologic cascade in STS. Previous studies have suggested cigarette exposure affects a wide range of host defense mechanisms, including decreased cytotoxic T cell activity and increased inflammatory response [23–26]. Inflammation from smoking increases mutations and silences tumor suppressor genes through various stress mechanisms. Smoking also promotes angio- and lymphangiogenesis, thereby increasing potential for tumor spread [23].

Lu et al. showed that cigarette smoke exposure increased the incidence of lung metastases following a B16-MO5 melanoma tumor challenge [27]. This study also showed that the carcinogens from cigarette smoke alter the immunity associated with increased tumor load by impairment of NK cell-dependent tumor immune surveillance [27]. Moreover, smokers have been shown to have decreased number and proportion of circulating NK cells, an effect seen in current smokers and those who report smoking cessation for more than 20 years [28]. This evidence suggests the complete restoration of NK cell levels following cessation appears to be profoundly suppressed or irreversible. Due to the myriad effects of cigarette smoke, it is conceivable NK cells are only one of many contributory factors of disease evolution.

Inflammatory mediators such as direct carcinogens, toxins, and oxidative compounds found in cigarette smoke are well established to activate nuclear factor-kappa B (NFκB), inducing expression of several growth factors that can increase tumor cell proliferation, survival, and migration through the phosphoinositide 3 kinase (PI3K)-Akt pathway [29]. Inflammatory macrophages, neutrophils, and mast cells produce extracellular matrix-degrading proteases and other factors that enable invasion and migration [30]. Thus, patients who had a prolonged exposure to smoke carcinogens likely had decreased immunity and dysregulated molecular signaling, leading to distant metastasis and disease progression as seen in this investigation.

This study also found that larger tumors (≥ 10 cm), age at diagnosis, tumor grade, and synovial sarcoma histology significantly impacted OS, DMFS, and PFS on MVA. Several studies have corroborated these findings, possibly having prognostic significance for STS independent of cohort subtype composition and patient differences. Increased tumor size (per 1 cm) predicted sarcoma-specific death and distant metastasis, but was not significant for local recurrence [18]. Others have demonstrated increasing size as a prognostic indicator for survival and distant metastasis [19, 31]. Age > 50 is associated with decreased postmetastatic survival [31] and sarcoma-specific death, but not distant metastasis or local recurrence [18, 32]. Another survival predictor, tumor grade, is well known to influence oncologic outcomes, as patients with high grade disease and a metastatic presentation have a poorer prognosis [31, 33]. Coindre et al. found patients with grade 3 tumors had poorer OS, along with an increased rate of local recurrence and distant metastasis [32]. Others have shown similar findings as higher tumor grade contributes to increased likelihood of distant metastasis and mortality [34–37]. Interestingly, histological diagnosis of synovial sarcoma was associated with a 13-fold increased risk of death when compared with other sarcomas after prognostic factors had been accounted for [13]. Tumor size (> 5 cm), non-extremity location, metastatic disease on initial presentation, and monophasic subtype was associated with poorer overall survival in synovial sarcoma; monophasic subtype was also associated with poorer PFS [38]. Further investigations have documented synovial sarcoma to represent a poor prognosis compared with other STS histologies [39, 40].

Limitations to this study include a modest cohort size and the heterogeneous sarcoma profile contained herein. While follow-up time was limited, several confounding variables can alter the OS of STS patients. Therefore, any effect of smoking on OS may take years to determine. Another limitation was the retrospective nature of our study which inherently introduced biases as a byproduct of this design. Smoking history was also represented as a dichotomous term, narrowing our intra-study variables. Other limitations include potential bias of selecting only pre-operative RT and chemotherapy/RT patients, although this is the general recommendation from our center. Additionally, history was gathered under the assumption that the self-reporting of smoking history was accurate. Future investigations should consider gathering a comprehensive smoking history including: pack years, use of other tobacco products, use of nicotine replacement therapy, and length of abstinence (if applicable).

## Conclusion

Current and former smoking is associated with DMFS and PFS in STS patients treated with pre-operative RT. Smoking may lead to immunologic compromise, likely increasing the potential for tumor spread. A complete smoking history may allow physicians to better predict survival rates, and as such, should be gathered in future cohort studies as a potential correlative variable and prognostic indicator. Additional information regarding the extent of smoking may further delineate the role smoking has on the prognosis of STS patients.

## Abbreviations

CI: confidence interval
CRT: chemoradiotherapy
DMFS: distant metastasis-free survival
Gy: gray
IMRT: intensity-modulated RT
KPS: Karnofsky performance status
MVA: multivariate analysis
NFκB: nuclear factor-kappa B
OS: overall survival
PFS: progression-free survival
PI3K: phosphoinositide 3 kinase
RT: radiation
STS: soft tissue sarcoma
UVA: univariate analysis
